# Discrimination and Mental Health During the Black Lives Matter Movement and the COVID-19 Pandemic

**DOI:** 10.1093/geroni/igab046.1858

**Published:** 2021-12-17

**Authors:** Peiyi Lu, Dexia Kong, Mack Shelley

**Affiliations:** 1 Columbia University, Fort Lee, New Jersey, United States; 2 The Chinese University of Hong Kong, Hong Kong , Hong Kong; 3 Iowa State University, Ames, Iowa, United States

## Abstract

Discrimination has been more prevalent since the pandemic. Black Lives Matter (BLM) movement flourished in the summer of 2020 as protests against police brutality and racial injustice. However, the extent to which individuals’ discrimination experiences and associated mental health outcomes change amid a global pandemic and a dramatic societal movement in American society remains unknown. This study examines the dynamic relationship of racism and/or Covid-19-related discrimination with changes in mental health in the context of BLM and Covid-19. Data were from U.S. adults (age ≥18) who completed the online Understanding Coronavirus in America survey in June and September of 2020 (n=3,502). Respondents were asked to attribute their discrimination experience to 8 main reasons: (1) Covid-19; (2) national origin/race/skin color; (3) gender/sexual orientation; (4) age; (5) physical feature; (6) education/income; (7) health condition; and (8) religion/other. Quasi-Poisson regression models examined the associations between discrimination and anxiety/depression/stress. Results indicated about 33% of respondents reported discrimination in June compared to 21% in September. Racism was significantly associated with more anxiety/depression/stress in June, but not in September or in the longitudinal trend. Covid-19-related discrimination was associated with elevated levels of anxiety/depression/stress in September and in the longitudinal trend, but not in June 2020. We concluded that individuals’ discrimination experiences are shaped by societal contexts. Specifically, racism was the dominant discrimination attribution in June 2020 when BLM was at its peak. However, Covid-19 discrimination overtook racism as the primary attribution and showed a significant relationship with poorer mental health over time.

